# The Essential Oil from *Conyza bonariensis* (L.) Cronquist (Asteraceae) Exerts an In Vitro Antimelanoma Effect by Inducing Apoptosis and Modulating the MAPKs, NF-κB, and PKB/AKT Signaling Pathways

**DOI:** 10.3390/ph16111553

**Published:** 2023-11-02

**Authors:** Rafael Carlos Ferreira, Sâmia Sousa Duarte, Valgrícia Matias de Sousa, Ramon Ramos Marques de Souza, Karinne Kelly Gadelha Marques, Renata Albuquerque de Abrantes, Yuri Mangueira do Nascimento, Natália Ferreira de Sousa, Marcus Tullius Scotti, Luciana Scotti, Josean Fechine Tavares, Juan Carlos Ramos Gonçalves, Marcelo Sobral da Silva, Marianna Vieira Sobral

**Affiliations:** Postgraduate Program in Natural Products and Bioactive Synthetics, Federal University of Paraíba, João Pessoa 58051-970, PB, Brazilramonramos.rm@gmail.com (R.R.M.d.S.); karinnegadelha@hotmail.com (K.K.G.M.);

**Keywords:** natural products, essential oil, antitumor effect

## Abstract

The characterization and cytotoxicity of the essential oil from *Conyza bonariensis* (L.) aerial parts (CBEO) were previously conducted. The major compound was (*Z*)-2-lachnophyllum ester (EZ), and CBEO exhibited significant ROS-dependent cytotoxicity in the melanoma cell line SK-MEL-28. Herein, we employed the Molegro Virtual Docker v.6.0.1 software to investigate the interactions between the EZ and Mitogen-Activated Protein Kinases (MAPKs), the Nuclear Factor *kappa* B (NF-κB), and the Protein Kinase B (PKB/AKT). Additionally, in vitro assays were performed in SK-MEL-28 cells to assess the effect of CBEO on the cell cycle, apoptosis, and these signaling pathways by flow cytometry and the 3-(4,5-dimethylthiazol-2-yl)-2,5-diphenyltetrazolium bromide (MTT) assay using MAPKs inhibitors. CBEO induced a significant increase in the sub-G1 peak, as well as biochemical and morphological changes characteristic of apoptosis. The in-silico results indicated that EZ interacts with Extracellular Signal-Regulated Kinase 1 (ERK1), c-Jun N-terminal Kinase 1 (JNK1), p38α MAPK, NF-κB, and PKB/AKT. Moreover, CBEO modulated the ERK1/2, JNK, p38 MAPK, NF-κB, and PKB/AKT activities in SK-MEL-28 cells. Furthermore, CBEO’s cytotoxicity against SK-MEL-28 cells was significantly altered in the presence of MAPKs inhibitors. These findings support the in vitro antimelanoma effect of CBEO through apoptosis induction, and the modulation of ERK, JNK, p38 MAPK, NF-κB, and PKB/AKT activities.

## 1. Introduction

Cutaneous melanoma is the most aggressive type of skin cancer [1], originating from the malignant transformation of melanocytes [2]. This cancer represents a global public health problem, with 57,000 recorded deaths in 2020 [3].

Chemotherapy remains a significant pharmacological modality for the treatment of many cancer types [4], including melanoma [5]. However, issues are associated with antineoplastic chemotherapy, such as chemoresistance [6,7,8]. Therefore, research should be conducted to obtain new therapeutic agents [9]. In this context, natural products standout as a valuable source of molecules with potential applications for cancer treatment [10].

**Figure 1:**
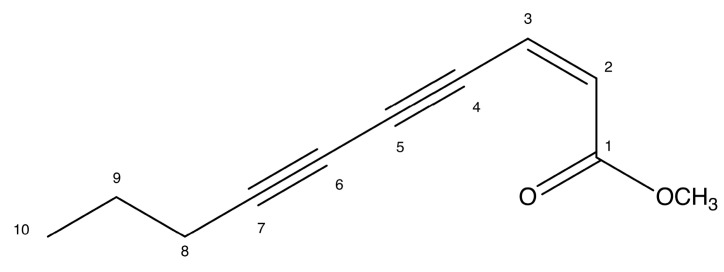
Molecular structure of (*Z*)-2-lachnophyllum ester (EZ).

Essential oils (EOs) are oily and volatile liquids [11], isolated from various parts of plants such as bark, flowers, and leaves [12,13]. The components of EOs include terpenes, terpenoids, esters, and aromatic phenols [14,15]. *Conyza bonariensis* (L.) Cronquist (Asteraceae) is a common weed in South America [16]. EOs from *Conyza bonariensis* exhibit various biological activities, such as antitumor effects [17,18,19]. Ferreira et al. (2023) [19] obtained an unprecedented EO from *Conyza bonariensis* aerial parts (CBEO) cultivated in João Pessoa, PB, Brazil, and collected in 2019. The CBEO was obtained by hydrodistillation using a Clevenger-type apparatus and characterized by Gas Chromatography-Mass Spectrometry (GC-MS). The chemical characterization of CBEO allowed the identification of 96.95% of its components (Figures S1 and S2). Approximately 39% of its constituents were monoterpenes and sesquiterpenes (39.71%), such as β-sesquiphellandrene (7.04%) and limonene (14.26%). The major compound detected was (Z)-2-lachnophyllum ester (EZ) (57.24%), an acetylenic compound (Figure 1). Furthermore, CBEO showed significant cytotoxicity against human tumor cell lines, particularly human melanoma cells SK-MEL-28 (half maximal inhibitory concentration, IC_50_ = 18.65 ± 1.16 µg/mL). Finally, these authors observed that the in vitro antimelanoma effect of CBEO is dependent on the generation of the Reactive Oxygen Species (ROS).

The antimelanoma effect of various EOs and some of their components occurs through multiple mechanisms, including cell cycle arrest and the induction of apoptosis [20]. Additionally, it has been demonstrated that the increase in ROS is involved in the antitumor effect by modulating intracellular signaling pathways, including the Mitogen-Activated Protein Kinases (MAPKs) [21], Nuclear Factor *kappa* B (NF-κB) [22], and Protein Kinase B (PKB/AKT) [23].

Apoptosis, or programmed cell death type I, involves a series of tightly controlled events. Cells undergoing apoptosis exhibit biochemical and morphological characteristics, such as DNA condensation and fragmentation, and the exposure of phosphatidylserine on the outer surface of the plasma membrane [24]. During carcinogenesis, cells acquire the ability to evade apoptosis, resulting in proliferative and survival advantages [25].

Mitogen-activated protein kinases (MAPKs) include Extracellular Signal-Regulated Kinases 1 and 2 (ERK1 and ERK2); c-Jun N-terminal Kinases 1, 2, and 3 (JNK1, JNK2, and JNK 3); and p38 Mitogen-Activated Protein Kinases α, β, γ, and δ (p38α, p38β, p38γ, and p38δ) [26]. Regarding the involvement of the MAPKs’ signaling pathways in cancer, it is known that approximately 50% of melanomas exhibit mutations in the *BRAF* oncogene, leading to constitutive activation of the Extracellular Signal-Regulated Kinases 1 and 2 (ERK1/2) pathway and uncontrolled proliferation [27]. Zhou et al. (2021) [28] observed that the expression of utrophin, a protein encoded by the tumor suppressor gene UTRN, is reduced in the human melanoma cell line A375, and its antiproliferative activity in these cells involves the inhibition of the c-Jun N-terminal Protein Kinase (JNK) pathway. In *BRAF* mutant melanoma cells (A375 and SK-MEL-28), the activation of p38 MAPK is associated with the negative regulation of subtype 4b plasma membrane Ca^2+^ pumps (PMCA4b), a metastasis suppressor [29]. However, it has been reported that these intracellular signaling pathways have pleiotropic roles. In fact, several clinically used antineoplastic drugs, such as doxorubicin, induce the activation of ERK1/2 and p38 MAPK [30,31,32]. Additionally, in ovarian carcinoma cells, the activation of autophagy-dependent cell death can be induced by the positive modulation of JNK [33].

The NF-κB family consists of five transcription factors that form homo- or heterodimers: c-Rel, RelA (p65), RelB, NF-κB1 (p105/p50), and NF-κB2 (p100/p52) [34], all possessing a Rel homology domain (RHD) for DNA binding [35]. In cancer, the NF-κB signaling pathway contributes to tumor progression by activating the transcription of multiple genes, such as those involved in apoptosis inhibition and angiogenesis induction [36]. On the other hand, it has also been reported that the cytotoxicity of some antitumor drugs depends on the activation of this pathway [37,38,39].

The PKB/AKT protein signaling pathway plays essential roles in cellular physiology, including growth, survival, proliferation, and metabolism [40]. Various diseases are associated with the dysregulation of this signaling pathway, such as diabetes and cardiovascular and neurological disorders [41]. In cancer, the overexpression and/or aberrant activation of proteins in this pathway are linked to the development of various malignant tumors. Constitutive activation of PKB/AKT is present in approximately 70% of melanomas [42,43]. Conversely, Sun et al. (2019) [44] demonstrated the involvement of the activation of the PKB/AKT pathway in the antitumor effect through the induction of apoptosis by aloin and metformin, alone or in combination, in liver cancer models.

Therefore, considering that cell cycle arrest, apoptosis induction, and the modulation of the MAPK, NF-κB, and PKB/AKT signaling pathways are promising targets for cancer treatment, we present here the in vitro effects of CBEO on the cell cycle and the induction of apoptosis. Additionally, we investigated the molecular docking prediction between the major compound of CBEO, EZ, and the proteins ERK1, JNK1, p38α MAPK, NF-κB (p50/p65), and PKB/AKT. Finally, our study provides insight into the in vitro effect of CBEO on the modulation of MAPK, NF-κB, and PKB/AKT proteins in the human melanoma cell line SK-MEL-28.

## 2. Results

### 2.1. CBEO Treatment Increases the Percentage of Cells in the Sub-G1 Peak

**Figure 2:**
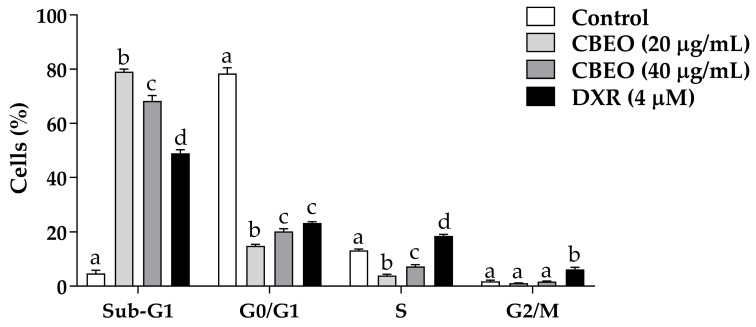
Percentage of cells in the different phases of the cell cycle and at the sub-G1 peak after 48 h of treatment with essential oil from *Conyza bonariensis* (L.) aerial parts (CBEO) (20 or 40 µg/mL) or doxorubicin (DXR) (4 µM). Data are obtained from three independent experiments carried out in duplicate and expressed as mean ± standard error of the mean (SEM) analyzed by Analysis of Variance (ANOVA) one-way followed by Tukey’s test. Different letters denote significant differences between experimental groups for each condition; *p* < 0.05.

CBEO treatment induced a significant increase in the percentage of cells in the sub-G1 peak (20 µg/mL: 79.09 ± 0.98%; 40 µg/mL: 68.25 ± 2.06%, *p* < 0.05 for all), compared to the control (4.51 ± 1.31%). Doxorubicin (DXR) induced a significant increase in the percentage of cells in the S phase (18.57 ± 0.59%, *p* < 0.05) and G2/M phase (6.17 ± 0.80%, *p* < 0.05), compared to the control (S phase: 13.18 ± 0.54%; G2/M phase: 1.80 ± 0.42%). Furthermore, DXR treatment increased the percentage of cells in the sub-G1 peak (48.94 ± 1.31%, *p* < 0.05) (Figure 2).

### 2.2. CBEO Treatment Induces Apoptosis

**Figure 3:**
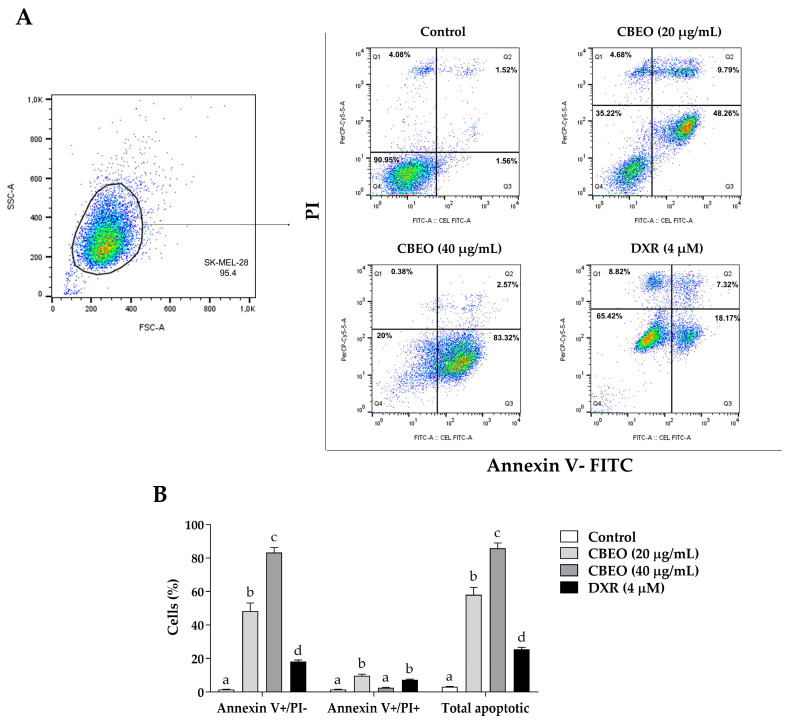
Effect of the essential oil from *Conyza bonariensis* (L.) aerial parts (CBEO) and doxorubicin (DXR) on human melanoma SK-MEL-28 cells labeled with annexin V–fluorescein isothiocyanate (FITC) and/or propidium iodide (PI) after 48 h of treatment. (**A**) Representative dotplots were obtained through a flow cytometry analysis. The dotplots were divided into four quadrants representing different cellular populations: viable cells (annexin V–FITC-/PI−, lower left quadrant), cells in early apoptosis (annexin V–FITC+/PI−, lower right quadrant), cells in late apoptosis/necrosis (annexin V–FITC+/PI+, upper right quadrant), and dead cells (annexin V–FITC−/PI+, upper left quadrant). A total of 10,000 events/samples were acquired using red fluorescence detectors (PI, 325–488 nm) and green fluorescence detectors (FITC, 493–525 nm). (**B**) Percentage of cells labeled with annexin V–FITC and/or PI. Data were obtained from three independent experiments performed in duplicate and analyzed by Analysis of Variance (ANOVA) one-way followed by Tukey’s test. Different letters indicate significant differences between experimental groups for each condition. *p* < 0.05.

CBEO treatment induced a significant increase in the percentage of cells in early apoptosis (annexin V–FITC+/PI−) (20 µg/mL: 48.26 ± 4.83%; 40 µg/mL: 83.32 ± 2.97%, *p* < 0.05 for both) and late apoptosis/necrosis (annexin V–FITC+/PI+) (20 µg/mL: 9.79 ± 0.94%, *p* < 0.05) compared to the control (early apoptosis: 1.56 ± 0.16%; late apoptosis/necrosis: 1.53 ± 0.15%, respectively). Therefore, CBEO treatment significantly increased the percentage of apoptotic cells (total apoptosis) (20 µg/mL: 58.05 ± 4.41%; 40 µg/mL: 85.89 ± 3.17%, *p* < 0.05 for both) compared to the control (3.09 ± 0.27%). DXR induced a significant increase in the number of cells in early apoptosis (18.17 ± 0.84%, *p* < 0.05), late apoptosis/necrosis (7.32 ± 0.34%, *p* < 0.05), and total apoptotic cells (25.49 ± 1.14%, *p* < 0.05) (Figure 3).

### 2.3. CBEO-Treated Cells Exhibit Morphological Characteristics of Apoptosis in Confocal Microscopy

**Figure 4:**
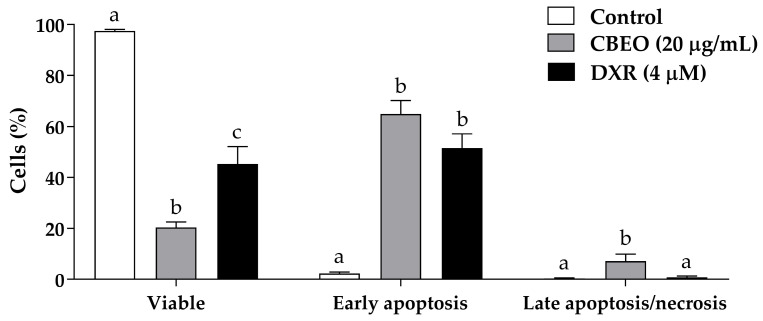
Apoptosis induction by the essential oil from *Conyza bonariensis* (L.) aerial parts (CBEO) or doxorubicin (DXR) analyzed through acridine orange (AO) and propidium iodide (PI) staining in human melanoma SK-MEL-28 cells after 48 h of treatment. Data were obtained from three independent experiments carried out in duplicate and expressed as mean ± standard error of the mean (SEM) analyzed by Analysis of Variance (ANOVA) one-way followed by Tukey’s test. Different letters denote significant differences between experimental groups for each condition; *p* < 0.05.

Figure 4 shows SK-MEL-28 cells stained with acridine orange (AO) and/or propidium iodide (PI) and analyzed by confocal microscopy. CBEO treatment (20 µg/mL) induced a significant increase in the percentage of cells with typical characteristics of early apoptosis (64.96 ± 5.28%, *p* < 0.05) and late apoptosis/necrosis (12.65 ± 5.85%, *p* < 0.05) compared to the control (early apoptosis: 2.40 ± 0.55%; late apoptosis/necrosis: 0.39 ± 0.16%, respectively). DXR treatment significantly increased the percentage of cells in early apoptosis (51.56 ± 5.59%, *p* < 0.05) compared to the control.

**Figure 5:**
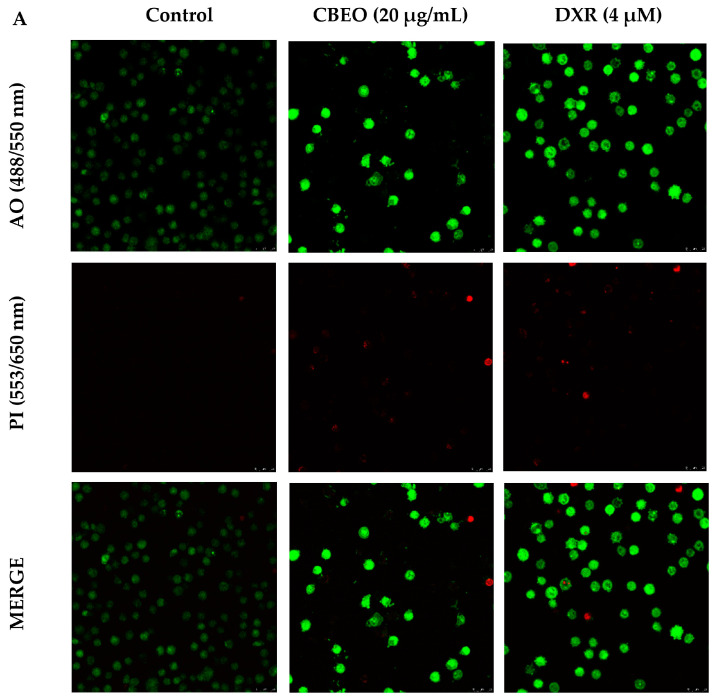

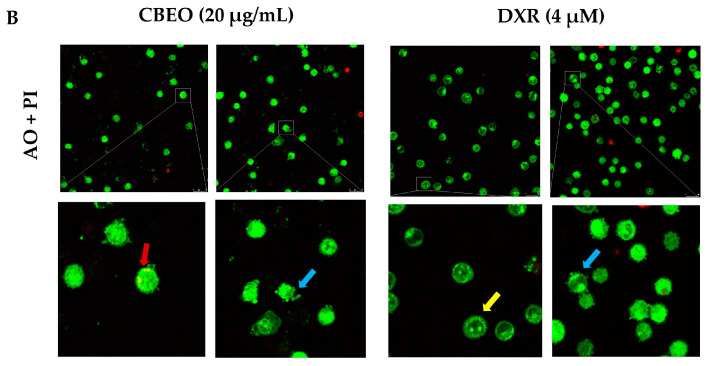
Representative images of SK-MEL-28 cells after 48 h of treatment with essential oil from *Conyza bonariensis* (L.) aerial parts (CBEO) (20 µg/mL) or DXR (4 μM), stained with acridine orange (AO) and/or propidium iodide (PI). (**A**) Viable cells exhibit a light green nucleus and an intact structure. Early apoptotic cells display a light green nucleus showing chromatin condensation. Late apoptotic cells show dense orange areas (green/red) of chromatin condensation and membrane blebs, while necrotic cells have a red nucleus. (**B**) Representative enlarged images of SK-MEL-28 cells after 48 h of incubation with CBEO (20 µg/mL) or DXR (4 μM). The yellow arrow indicates chromatin condensation, the blue arrow indicates membrane blebs, and the red arrow indicates DNA fragmentation.

In addition, representative images of cells treated with CBEO (20 µg/mL) or DXR, which exhibit characteristics of early and late apoptosis, are shown in Figure 5.

### 2.4. Docking Prediction

The molecular docking prediction between (Z)-2-lachnophyllum ester (EZ) or other ligands and Extracellular Signal-Regulated Kinase 1 (ERK1), c-Jun N-terminal Kinase 1 (JNK1), p38 Mitogen-Activated Protein Kinase α (p38α MAPK), Nuclear Factor *kappa* B (NF-κB; p50/p65), and Protein Kinase B (PKB/AKT) were generated using two scoring functions: the Moldock score and the Rerank Score. More negative values indicated better predictions for all scoring functions.

Before carrying out the molecular docking simulations, the validation of the compounds under study was carried out through redocking between the ligands and cocrystallized proteins. In redocking, the RMSD (Root Mean Square Deviation) value is determined. This value is calculated by comparing the coordinates of the heaviest atoms in the experimentally determined crystallographic structure with their counterparts in the docked position. An RMSD value of up to 2 Å is considered acceptable for this metric.

The redocking calculation was performed only for enzymes that present a cocrystallized ligand and corresponded to 1.2477 for the ERK1 in the complex with the ligand [(1R,4Z)-cyclooct-4-en-1-yl]-N-[4-[4-[[5-chloro-4-[[2-(propanoylamino)phenyl]amino]pyrimidin-2-yl]amino]pyridin-2-yl]but-3-ynyl]carbamate (PDB: 5LCJ) and 0.1135 for the JNK1 in the complex with the ligand 6-chloro-9-hydroxy-1,3-dimethyl-1,9-dihydro-4H-pyrazolo(3,4-b)quinolin-4-one (PDB: 2G01). During the redocking analysis, it was observed that the RMSD value was less than 2.0 Å, indicating that the generated pose correctly positioned the ligand within the active site. This indicates that the program provided values considered satisfactory for the docking validation. The docking results for the targets under study, according to the energy score of the MolDock Score algorithm, can be viewed in Table 1.

**Figure 6:**
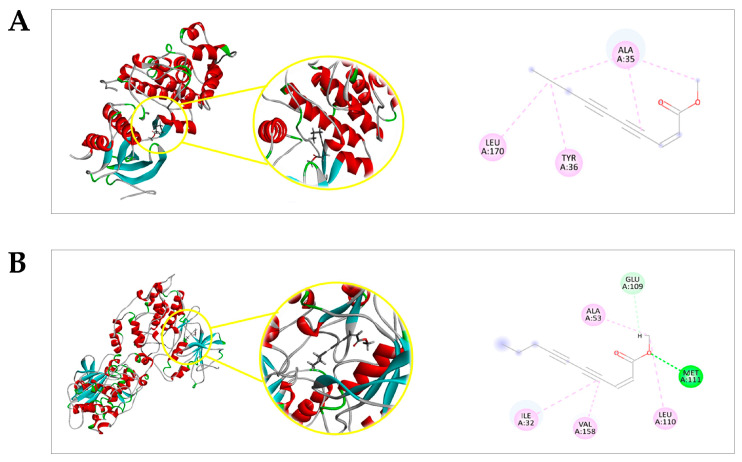

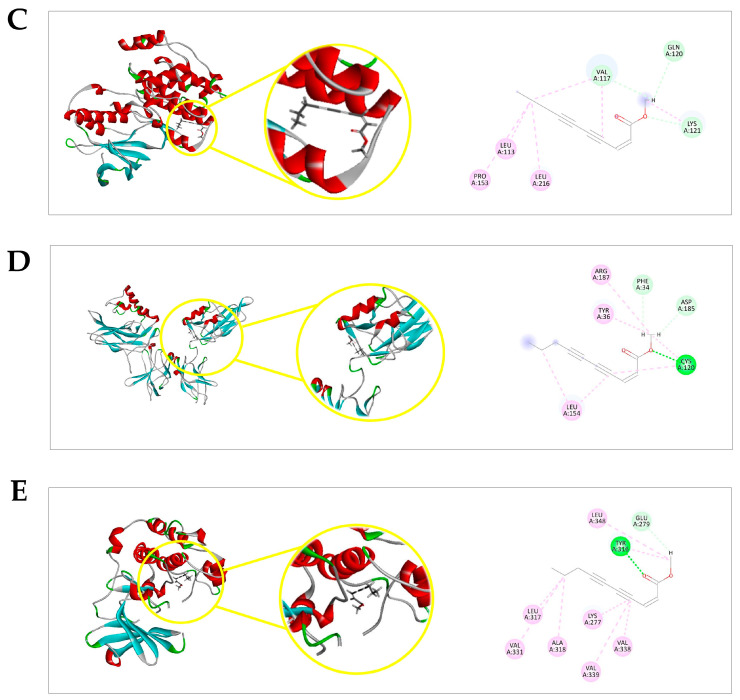
PDB ID protein—(*Z*)-2-lachnophyllum ester (EZ) interactions. (**A**) Maps of interactions between the PDB protein ID 5LCJ (ERK1) and EZ; (**B**) Maps of interactions between the PDB protein ID 2G01 (JNK1) and EZ; (**C**) Maps of interactions between the PDB protein ID 1R39 (p38α MAPK) and EZ; (**D**) Maps of interactions between the PDB protein ID 1VKX (NF-κB; p50/p65) and EZ; (**E**) Maps of interactions between the PDB protein ID 1GZN (PKB/AKT) and EZ.

EZ presented negative ligand–receptor interaction energies, and displayed affinity probability values above 0.60 with ERK1 (*p* = 0.6405279), JNK1 (*p* = 0.6692), p38α MAPK (*p* = 0.863618), NF-κB (p50/p65) (*p* = 0.8910), and PKB/AKT (*p* = 0.766702). Figure 6 shows that EZ interactions with the amino acid residues are mediated by hydrogen bond interactions (dashed green lines), hydrophobic interactions (dashed pink lines: π-alkyl; dashed purple lines: π-σ; dashed blue lines: halogens—Br, Cl, and I), and/or steric interactions (dashed red lines: donor-donor; dashed orange lines: π-anion).

### 2.5. CBEO Treatment Modulates the Activity of MAPKs (Mitogen-Activated Protein Kinases)

**Figure 7:**
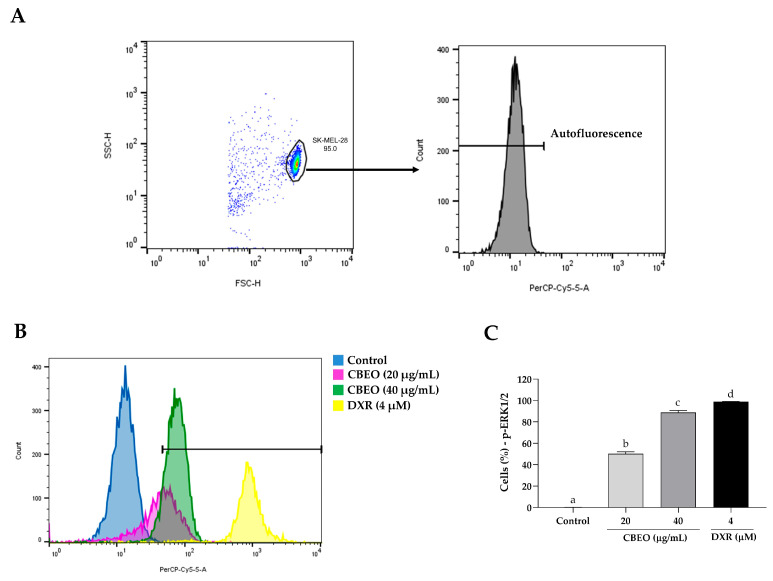
Extracellular Signal-Regulated Kinase 1 and 2 (ERK1/2) modulation in SK-MEL-28 cells after treatment with essential oil from *Conyza bonariensis* aerial parts (CBEO) (20 or 40 μg/mL) or DXR (4 µM). (**A**) The cell population was determined from the analysis of cell size dotplots (FSC–Forward Scatter, *X*-axis) and cytoplasmic granularity (SSC–Side Scatter, *Y*-axis) obtained by flow cytometry. For the analysis of ERK1/2 fluorescence histograms (PerCP-Cy5.5), a region corresponding to the autofluorescence of cells not labeled with the antibody was initially delimited. Only the percentage of fluorescent cells after this demarcated region was considered positive. (**B**) Overlay of representative histograms showing ERK1/2 fluorescence (PerCP-Cy5.5, *X*-axis) and the number of cells/events (*Y*-axis) in different experimental groups. (**C**) Graphical representation of the results obtained by flow cytometry. Data were obtained from three independent experiments carried out in duplicate and expressed as mean ± standard error of the mean (SEM) analyzed by Analysis of Variance (ANOVA) one-way followed by Tukey’s test. Different letters denote significant differences among conditions; *p* < 0.05.

CBEO treatment induced an increase in the percentage of cells labeled with anti-ERK1/2 (20 µg/mL: 34.70 ± 1.37%; 40 µg/mL: 74.46 ± 3.44%, *p* < 0.05 for both), compared to the control (0.09 ± 0.01%). In addition, DXR treatment induced a significant increase in the percentage of cells labeled with anti-ERK1/2 antibodies (98.87 ± 0.16%, *p* < 0.05), compared to the control (Figure 7).

**Figure 8:**
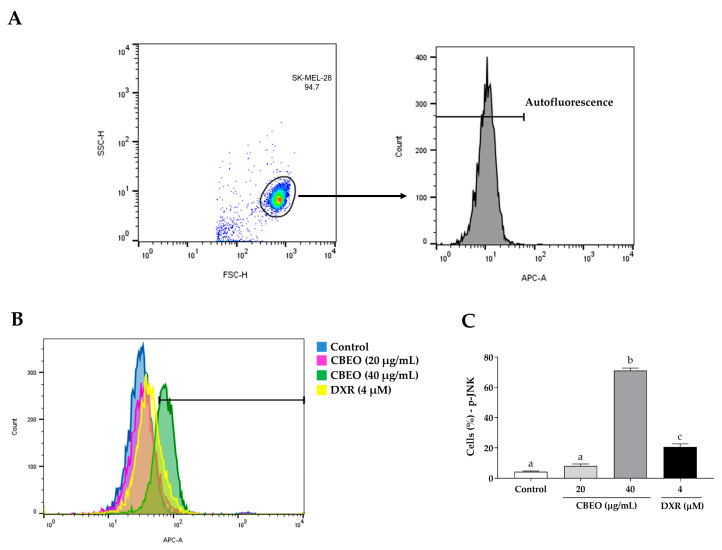
c-Jun N-terminal Kinase (JNK) modulation in SK-MEL-28 cells after treatment with essential oil from *Conyza bonariensis* aerial parts (CBEO) (20 or 40 μg/mL) or DXR (4 µM). (**A**) The cell population was determined from the analysis of cell size dotplots (FSC–Forward Scatter, *X*-axis) and cytoplasmic granularity (SSC–Side Scatter, *Y*-axis) obtained by flow cytometry. For the analysis of JNK fluorescence histograms (APC), a region corresponding to the autofluorescence of cells not labeled with the antibody was initially delimited. Only the percentage of fluorescent cells after this demarcated region was considered positive. (**B**) Overlay of representative histograms showing JNK fluorescence (APC, *X*-axis) and the number of cells/events (*Y*-axis) in different experimental groups. (**C**) Graphical representation of the results obtained by flow cytometry. Data were obtained from three independent experiments carried out in duplicate and expressed as mean ± standard error of the mean (SEM) analyzed by Analysis of Variance (ANOVA) one-way followed by Tukey’s test. Different letters denote significant differences among conditions; *p* < 0.05.

As observed in Figure 8, CBEO treatment induced an increase in the percentage of cells labeled with anti-JNK antibodies (40 µg/mL: 61.20 ± 1.90%, *p* < 0.05), compared to the control (2.82 ± 0.27%). The standard drug DXR also induced a significant increase in the percentage of cells labeled with anti-JNK antibodies (16.19 ± 1.33%, *p* < 0.05), compared to the control.

**Figure 9:**
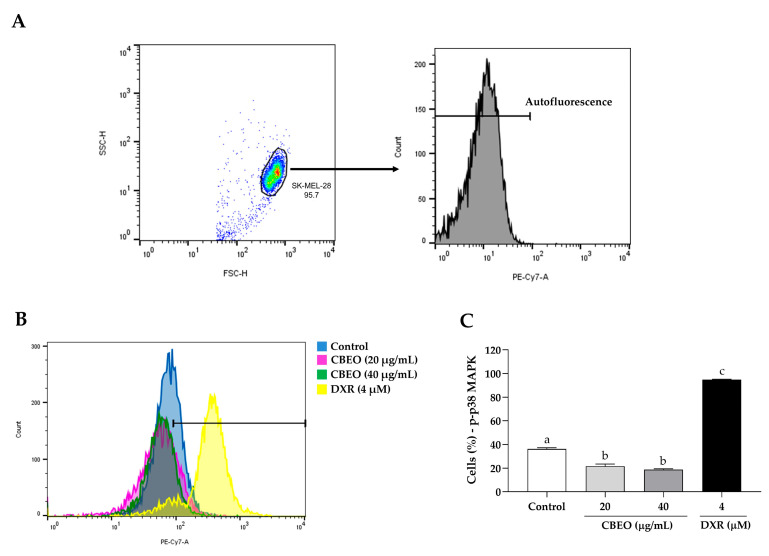
p38 Mitogen-Activated Protein Kinase (p38 MAPK) modulation in SK-MEL-28 cells after treatment with essential oil from *Conyza bonariensis* aerial parts (CBEO) (20 or 40 μg/mL) or DXR (4 µM). (**A**) The cell population was determined from the analysis of cell size dotplots (FSC–Forward Scatter, *X*-axis) and cytoplasmic granularity (SSC–Side Scatter, *Y*-axis) obtained by flow cytometry. For the analysis of p38 MAPK fluorescence histograms (PE-Cy7), a region corresponding to the autofluorescence of cells not labeled with the antibody was initially delimited. Only the percentage of fluorescent cells after this demarcated region was considered positive. (**B**) Overlay of representative histograms showing p38 MAPK fluorescence (PE-Cy7, *X*-axis) and the number of cells/events (*Y*-axis) in different experimental groups. (**C**) Graphical representation of the results obtained by flow cytometry. Data were obtained from three independent experiments carried out in duplicate and expressed as mean ± standard error of the mean (SEM) analyzed by Analysis of Variance (ANOVA) one-way followed by Tukey’s test. Different letters denote significant differences among conditions; *p* < 0.05.

In contrast, CBEO treatment reduced the percentage of cells labeled with anti-p38 MAPK antibodies (20 µg/mL: 21.67 ± 1.92%; 40 µg/mL: 18.82 ± 0.72%, *p* < 0.05 for both), compared to the control (36.30 ± 1.00%). DXR treatment induced a significant increase in the percentage of cells labeled with anti-p38 MAPK antibodies (94.80 ± 0.45%, *p* < 0.05), compared to the control (Figure 9).

### 2.6. CBEO Cytotoxicity in SK-MEL-28 Cells Is MAPK-Dependent

**Figure 10:**
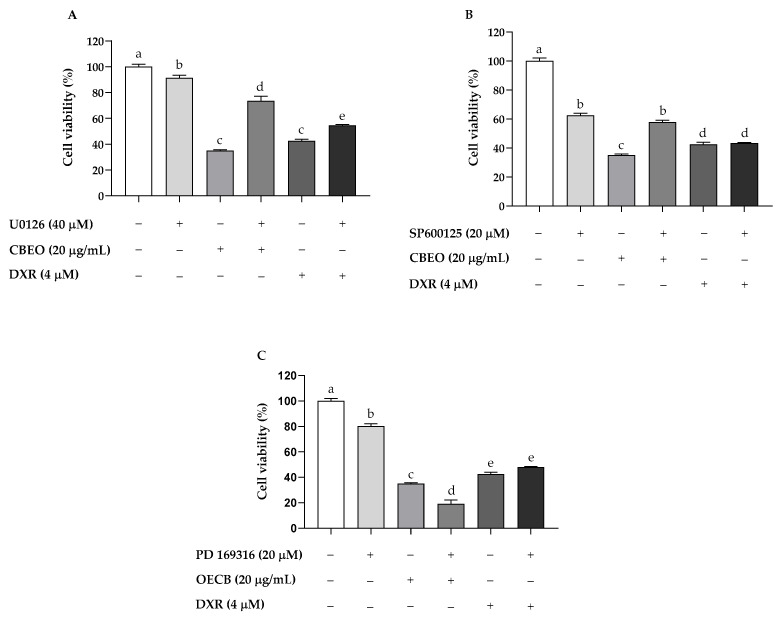
Percentual of cell viability after 72 h of essential oil from *Conyza bonariensis* (L.) aerial parts (CBEO) (20 µg/mL) or doxorubicin (DXR) (4 µM) treatment in the presence or absence of (**A**) ERK (U0126), (**B**) JNK (SP600125), or (**C**) p38 MAPK (PD 169316) inhibitors. Data were obtained from three independent experiments conducted in triplicate and analyzed by Analysis of Variance (ANOVA) one-way followed by Tukey’s test. Different letters denote significant differences between conditions. *p* < 0.05.

CBEO treatment (20 µg/mL) in the absence of MAPK inhibitors induced a significant reduction in cell viability (35.13 ± 0.65%, *p* < 0.05) compared to the control (100.00 ± 1.97%). ERK inhibitor (U0126, 40 μM) or JNK inhibitor (SP600125, 20 μM) pretreatment significantly prevented CBEO cytotoxicity (cell viability: 73.54 ± 3.68%; and 57.74 ± 1.41%, respectively; *p* < 0.05 for both) compared to the group treated with CBEO in the absence of MAPK inhibitors (Figure 10A,B). In contrast, p38 MAPK inhibitor (PD 169316, 20 μM) pretreatment significantly increased the CBEO cytotoxic effect (19.19 ± 2.94%, *p* < 0.05) compared to the group treated with CBEO in the absence of MAPK inhibitors (Figure 10C). For the standard drug DXR, ERK inhibitor pretreatment significantly prevented the cytotoxicity of this drug (cell viability: 54.54 ± 0.73%, *p* < 0.05) compared to the group treated with DXR in the absence of MAPK inhibitors (42.59 ± 1.37%) (Figure 10A).

### 2.7. CBEO Treatment Modulates the Activity of NF-κB (Nuclear Factor kappa B)

**Figure 11:**
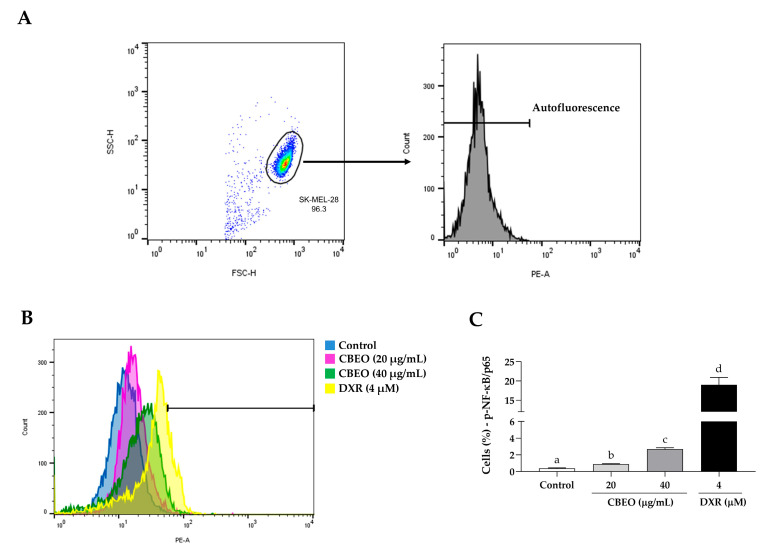
Nuclear Factor *kappa* B (NF-κB) modulation in SK-MEL-28 cells after treatment with essential oil from *Conyza bonariensis* aerial parts (CBEO) (20 or 40 μg/mL) or DXR (4 µM). (**A**) The cell population was determined from the analysis of cell size dotplots (FSC–Forward Scatter, *X*-axis) and cytoplasmic granularity (SSC–Side Scatter, *Y*-axis) obtained by flow cytometry. For the analysis of NF-κB fluorescence histograms (PE), a region corresponding to the autofluorescence of cells not labeled with the antibody was initially delimited. Only the percentage of fluorescent cells after this demarcated region was considered positive. (**B**) Overlay of representative histograms showing NF-κB fluorescence (PE, *X*-axis) and the number of cells/events (*Y*-axis) in different experimental groups. (**C**) Graphical representation of the results obtained by flow cytometry. Data were obtained from three independent experiments carried out in duplicate and expressed as mean ± standard error of the mean (SEM) analyzed by Analysis of Variance (ANOVA) one-way followed by Tukey’s test. Different letters denote significant differences among conditions; *p* < 0.05.

CBEO or DXR treatments induced a significant increase in the percentage of cells labeled with anti-NF-κB/p65 antibodies (CBEO 20 µg/mL: 0.90 ± 0.06%; CBEO 40 µg/mL: 2.69 ± 0.17%; DXR: 18.95 ± 1.94%, *p* < 0.05 for all), compared to the control (0.39 ± 0.05%) (Figure 11).

### 2.8. CBEO Treatment Modulates the Activity of PKB/AKT (Protein Kinase B)

**Figure 12:**
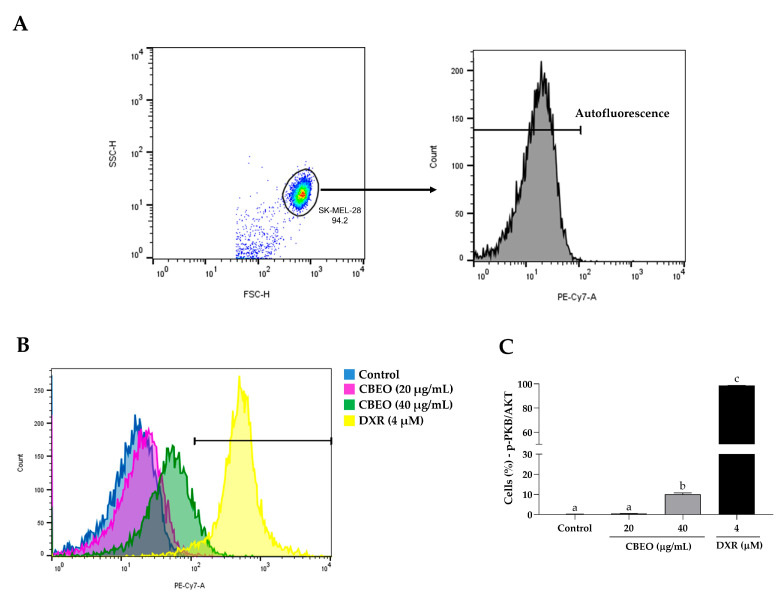
Protein Kinase B (PKB/AKT) modulation in SK-MEL-28 cells after treatment with essential oil from *Conyza bonariensis* aerial parts (CBEO) (20 or 40 μg/mL) or DXR (4 µM). (**A**) The cell population was determined from the analysis of cell size dotplots (FSC–Forward Scatter, *X*-axis) and cytoplasmic granularity (SSC–Side Scatter, *Y*-axis) obtained by flow cytometry. For the analysis of PKB/AKT fluorescence histograms (PE-Cy7), a region corresponding to the autofluorescence of cells not labeled with the antibody was initially delimited. Only the percentage of fluorescent cells after this demarcated region was considered positive. (**B**) Overlay of representative histograms showing PKB/AKT fluorescence (PE-Cy7, *X*-axis) and the number of cells/events (*Y*-axis) in different experimental groups. (**C**) Graphical representation of the results obtained by flow cytometry. Data were obtained from three independent experiments carried out in duplicate and expressed as mean ± standard error of the mean (SEM) analyzed by Analysis of Variance (ANOVA) one-way followed by Tukey’s test. Different letters denote significant differences among conditions; *p* < 0.05.

CBEO or DXR treatments induced a significant increase in the percentage of cells labeled with anti-PKB/AKT antibodies (CBEO 40 µg/mL: 10.03 ± 0.71%; DXR: 98.73 ± 0.12%, *p* < 0.05 for both), compared to the control (0.50 ± 0.01%) (Figure 12).

## 3. Discussion

Natural products represent many compounds currently used in antineoplastic chemotherapy. [45]. In this context, research has been conducted to investigate the antitumor effects of natural products, such as essential oils (EOs) [46]. The EO from *Conyza bonariensis* (L.) Cronquist aerial parts (CBEO) was previously obtained and characterized by Ferreira et al. (2023) [19]. The major compound identified was (*Z*)-2-lachnophyllum ester (EZ) (57.24%). CBEO exhibited toxicity in the zebrafish model, cytotoxicity in human peripheral blood mononuclear cells (PBMCs), and in vitro antitumor effects in the human melanoma cell line SK-MEL-28 by inducing oxidative stress. To better characterize the CBEO antimelanoma effect, we investigated its effect on cell cycle arrest, the induction of apoptosis, and the modulation of the Mitogen-Activated Protein Kinases (MAPKs), Nuclear Factor *kappa* B (NF-κB), and Protein Kinase B (PKB/AKT) signaling pathways through in silico and in vitro assays using the SK-MEL-28 cell line.

In our study, doxorubicin (DXR) was used as the standard drug. DXR is an anthracycline isolated from the species *Streptomyces peucetius* var. *caesius*, and it is still widely employed in antineoplastic chemotherapy, including the treatment of leukemias and breast cancer [47]. Its antitumor mechanism of action includes the inhibition of the enzyme topoisomerase II, the induction of cell cycle arrest, the induction of oxidative stress [48], and the modulation of the activity of Mitogen-Activated Protein Kinases (MAPKs) [49,50,51], nuclear factor *kappa* B (NF-κB) [52], and protein kinase B (PKB/AKT) [53].

The cell cycle is a tightly regulated process that leads to cell division. In cancer, this event becomes dysregulated, resulting in uncontrolled cell proliferation. Therefore, agents that interfere with the cell cycle are considered promising for antitumor chemotherapy [54]. The significant increase in the sub-G1 peak observed after CBEO treatment is characteristic of apoptotic cells [55]. This effect has also been observed after the exposure of tumor cells to other EOs [56,57,58]. Therefore, considering that the increase in the sub-G1 peak is related to apoptosis, the possible induction of apoptosis by CBEO was also investigated.

Apoptosis is a regulated process of cell death [59], characterized by cellular changes such as protein degradation, membrane blebbing, and DNA cleavage [60]. In cancer, cells exhibit resistance to apoptosis [61], and thus, the induction of this type of cell death has been a major goal in antitumor therapy [62]. CBEO induced an increase in the percentage of cells undergoing apoptosis. In addition, the analysis of the images obtained by laser confocal microscopy showed morphological changes characteristic of the apoptotic process, such as chromatin condensation, membrane blebs, and DNA fragmentation. Apoptosis is a shared mechanism of action for various EOs of the Asteraceae family. The EO from *Tridax procumbens* leaves induced apoptosis in murine melanoma B16-F10 cells and in the in vivo melanoma model [63]. Eos from *Artemisia arborescens* flowers and leaves were able to induce the apoptotic process [64]. *Carpesium abrotanoides’* EO induces apoptosis via the intrinsic pathway in human hepatocellular carcinoma cells (HepG2) [65]. Finally, the *Conyza canadensis* EO induced apoptosis in the human cervical cancer HeLa cell line [66]. Based on our results, CBEO induces an antimelanoma effect by apoptosis induction.

In melanoma, tumoral development is associated with oxidative stress, and Reactive Oxygen Species (ROS) are involved in the modulation of intracellular signaling pathways related to cell proliferation and apoptosis [21]. The ROS increase is involved in the antitumor effect of several drugs by modulating intracellular signaling pathways, including the MAPKs pathway [67], NF-κB pathway [22], and PKB/AKT pathway [23]. Ferreira et al. (2023) [19] observed that the CBEO antimelanoma effect is ROS-dependent. Therefore, as a preliminary analysis of the possible involvement of these intracellular signaling pathways in the antitumor effect of this EO, molecular docking was performed on crystallographic structures of these proteins to obtain protein-ligand complexes with the major compound, EZ, of CBEO.

MAPKs’ signaling pathways are involved in various cellular processes, including proliferation and survival [68]. In cancer, the dual role of these proteins has been reported. Therefore, stimulating the activity of these kinases can be involved in either a pro-tumoral or anti-tumoral effect [69,70]. A molecular docking analysis showed favorable interaction between EZ and the ERK1 protein through amino acid residues that also contribute to the interaction of this target with the standard drug DXR (Ala35) or the ligand from the Protein Data Bank (PDB) (Ala35 and Leu170). We did not find molecular docking data between ERK1 compounds structurally similar to EZ. Nevertheless, the literature reports such data for compounds from different chemical classes. Nagalakshmamma et al. (2021) [71] designed and synthesized compounds derived from 1,4-diisocyanatobenzene bisurea, namely 3a–3j. These compounds exhibited a significant in vitro antitumor effect against the HeLa cell line. In the molecular docking study, it was observed that the derivatives 3d and 3e, which were the most cytotoxic against the cell line used, had binding with the ERK protein through amino acid residues, including Ala35 and Leu170. EZ was also able to interact with the JNK1 protein for amino acid residues that are involved in the binding of DXR (Ala53, Ile32, and Val158) or the PDB ligand (Ala53, Ile32, Val158, and Met111) to this target. In addition, EZ showed a higher binding probability value compared to the PDB ligand. We did not find reports in the literature of in silico molecular interaction between JNK1 and compounds structurally similar to EZ. Sugara et al. (2021) [72] showed new haloxanthones, coded as 3CX, 10CX, 5BX, and 4BX, designed for potential antitumor activity and bound to JNK, involved amino acids that participated in the interaction between EZ and this protein (Ala53, Ile32, Val158, Leu110, Met111, and Glu109). Favorable binding was also observed between EZ and the p38α MAPK in Gln120 amino acid residue that is involved in the interaction of DXR with this protein. Similarly, Leu216 and Val117 involved in the binding of EZ are also involved in the interaction between p38α MAPK and (4-(4-fluorophenyl)-2-(4-methylsulfinylphenyl)-5-(4-pyridyl)-imidazole) (SB203580), a specific inhibitor of this protein [73]. We did not find molecular docking data between p38α MAPK and compounds structurally similar to EZ. Shankar et al. (2021) [74] investigated the interaction between agasthisflavone, a phytochemical compound found in *Anacardium occidentale* (Anacardiaceae), and p38α MAPK. These researchers observed that there is binding between agasthisflavone and p38α MAPK through the interaction with Pro153, which also participates in the binding between EZ and this protein.

NF-κB is a transcription factor that plays important roles in cellular functions such as proliferation, differentiation, and apoptosis [75]. Currently, there is substantial evidence pointing to the dual role of NF-κB in cancer [76]. There was a favorable interaction between EZ and NF-κB, with amino acid residues involved in this binding also participating in the interaction between DXR and NF-κB (Cys120, Arg187, and Tyr36). EZ showed a higher binding affinity to NF-κB when compared to the binding affinity of the ligand 4-methyl-N1-(3-phenylpropyl)benzene-1,2-diamine to this protein. We did not find molecular docking data between NF-κB and compounds structurally similar to EZ. Nevertheless, the literature reports such data for compounds from different chemical classes. Shiroma et al. (2020) [77], after a virtual screening, identified compound A55 (2-(3-carbamoyl-6-hydroxy-4-methyl-2-oxopyridin-1(2H)-yl)acetic acid) as a selective inhibitor of RelA(p65)-DNA binding. The molecular docking result showed that this compound interacted with the Tyr36 residue of NF-κB, which is also involved in the interaction between EZ and this protein target. Zazeri et al. (2020) [78], in the search for structural analogs of piperine and piperlongumine (PPL), which are alkaloids found in *Piper longum* [79] and known for their various biological activities including antitumor effects [80,81], obtained a series of new substances. In the in silico study, in addition to the natural compounds piperine and PPL, the synthesized compounds exhibited an interaction with NF-κB. The interaction between PPL and NF-κB involved all the residues that are also related to the interaction of EZ and this protein (Cys120, Arg187, Tyr36, Leu154, Phe34, and Asp185). It is known that the antitumor activity of PPL involves the modulation of NF-κB [82]. On the other hand, among the amino acids involved in the interaction between EZ and NF-κB, the interaction of piperine with this protein involves only the Arg187 residue. Among the analogs, compound 4a stood out, and its interaction with the molecular target involved residues Cys120, Arg187, Tyr36, Leu154, and Asp185.

The PKB/AKT protein regulates various cellular events, including cell proliferation and angiogenesis [83]. In cancer, its effect has been reported to be either pro-tumoral or antitumoral [44,84]. For this target, EZ showed significant interaction energy values, indicating a favorable binding. This interaction involved amino acid residues that also participate in the interaction between the ligand 8-[4-(1-aminocyclobutyl)phenyl]-9-phenyl-2H-[1,2,4]triazolo[3,4-f][1,6]naphthyridin-3-one (MK-2206) and this protein (Tyr316, Leu348, Leu317, Val331, Ala318, Lys277, Val338, and Glu279). As with the other molecular targets in this study, we did not find reports in the literature of an in silico molecular interaction between PKB/AKT and compounds structurally similar to EZ. Brevilin A, a molecule isolated from *Centipeda minima* (Asteraceae) with significant antitumor activity, interacts with PKB/AKT in amino acid residues such as Lys277 [85], which also belongs to the group of amino acids involved in the interaction between EZ and this target. This amino acid residue is also involved in the interaction between epirubicin, an anthracycline used in the treatment of cancers such as breast cancer [86], and PKB/AKT [87]. Another compound with significant antitumor activity, pelargonidin, found in red or pink fruits, interacts with PKB/AKT in residues that include Leu317 and Lys277 [88].

Therefore, our results demonstrate that the proteins ERK, JNK, p38 MAPK, NF-κB, and PKB/AKT are potential targets for investigating the antitumor mechanisms of CBEO. Thus, in vitro tests were conducted to assess the effect of this EO on the signaling pathways of these proteins.

SK-MEL-28 cell treatment with CBEO induced ERK and JNK activation, as evidenced by the increased percentage of fluorescent cells marked with anti-ERK and anti-JNK antibodies. Furthermore, corroborating that the CBEO antitumor effect involves the activation of these intracellular signaling pathways, the ERK inhibitor (U0126) and JNK inhibitor (SP600125) treatments partially prevented the cytotoxicity of this EO. This antitumor mechanism is also shared with other EOs. For instance, after treating human oral squamous carcinoma cells (KB) with the EO from *Artemisia lavandulaefolia* (Asteraceae) aerial parts, the induction of apoptosis was observed, related to the phosphorylation of ERK and the JNK increased [89]. Similarly, the EO from *Pinus densiflora* (Pinaceae) leaves induced apoptosis in human oral carcinoma cells (YD-8), involving ERK and JNK activation, ROS generation, and caspase activation [90]. Additionally, the EO from *Teucrium alopecurus* (Poaceae) leaves induced cytotoxicity in human colon cancer cells (HCT-116) through ERK and JNK activation [91], which were also observed in the in vitro antitumor effect of the EO from *Thymus hirtus* sp. *algeriensis* (Lamiaceae) aerial parts on this cell line [92]. Hence, when we consider that ROS have the potential to trigger ERK1/2 [32] and JNK activation [93], we can propose a connection between the apoptosis observed in SK-MEL-28 cells following CBEO treatment and the stimulation of ERK1/2 and JNK proteins. Furthermore, it appears that these proteins are influenced positively by the ROS generated as a result of EO treatment.

Regarding the p38 MAPK, CBEO reduced the percentage of cells marked with anti-p38 MAPK antibodies, and in the MTT assay, the p38 MAPK inhibitor (PD 169316) treatment significantly increased the CBEO cytotoxicity. The inhibition of p38 MAPK activity, which is involved in the antitumor effect of Eos, has also been reported in the literature. The antitumor effect of bergamot EOs, obtained from the *Citrus bergamia* (Rutaceae) fruit peel, on human neuroblastoma cell line SH-SY5Y, involves p38 MAPK inhibition [94]. Similarly, the EO from *Saussurea lappa* (Asteraceae) roots induced apoptosis in human hepatocellular carcinoma cell lines (SMMC-7721 and Hep3B) by concentration-dependent p38 MAPK inhibition [95]. Therefore, taking into account the dual role of p38 MAPK in cancer, we propose that the in vitro antimelanoma effect of CBEO is linked to its inactivation. Additionally, we suggest that the activation of JNK exerts a negative regulatory impact on p38 MAPK, as described in the literature [96].

The labeling of SK-MEL-28 cells with anti-NF-κB (p65) antibodies showed that this EO induces the activation of this protein. A similar effect was observed in KB cells after treatment with the EO from *Artemisia capillaris* (Asteraceae). In this study, cytosolic IκBα degradation increased nuclear translocation of the RelA (p65) protein as well as NF-κB binding to DNA [97]. Furthermore, isolated molecules from EOs have also demonstrated antitumor effects by inducing NF-κB activation. The limonene antitumor effect, one of the monoterpenes found in CBEO, in the murine lymphoma cell line BW5147, involved the increased of NF-κB nuclear translocation [98]. Additionally, patchouli alcohol isolated from the EO of *Pogostemon cablin* (Lamiaceae) exhibited significant cytotoxicity against HCT-116 and SW480 cells (human colon adenocarcinoma) by increasing NF-κB transcriptional activity. Moreover, a significant increase in p65 levels in the nucleus of HCT-116 cells was observed, confirming the involvement of the NF-κB pathway activation in the patchouli alcohol antitumor effect [99]. Therefore, it is conceivable that the activation of ROS-dependent apoptosis following the treatment of SK-MEL-28 cells with CBEO is contingent upon the activation of NF-κB. Furthermore, ROS may be associated with the activation of this protein, as described in the literature [100].

Finally, PKB/AKT activation was evident after CBEO treatment. This antitumor action mechanism was also observed for different fractions of the EO from *Boswellia sacra* (Burseraceae) gum resin in Panc-28 cells (human pancreatic adenocarcinoma). In this study, it was observed that the expression of phosphorylated PKB/AKT increased rapidly, peaking at 15 min after the EO from *Boswellia sacra* (Burseraceae) gum resin treatment. Subsequently, gradual decreases in this phosphorylated PKB/AKT expression were observed [101]. It has been demonstrated that the activation of PKB/AKT can be mediated by ROS [102]. Therefore, we propose that the activation of PKB/AKT is associated with the induction of oxidative stress induced by CBEO.

## 4. Materials and Methods

### 4.1. Drugs and Reagents

Dulbecco’s Modified Eagle’s Medium (DMEM) (Sigma-Aldrich^®^; St. Louis, MO, USA), Buffered phosphate solution (PBS) (Sigma-Aldrich^®^; St. Louis, MO, USA), doxorubicin (DXR) (Sigma-Aldrich^®^; St. Louis, MO, USA), penicillin–streptomycin (Sigma-Aldrich^®^; St. Louis, MO, USA), 3-(4,5-dimethylthiazol-2-yl)-2,5-diphenyltetrazolium bromide (MTT) (Sigma-Aldrich^®^; St. Louis, MO, USA), dimethylsulfoxide (DMSO) (Dinâmica^®^, Indaiatuba, SP, Brazil), Sodium Dodecyl Sulfate (SDS) (Êxodo Científica^®^, Sumaré, SP, Brazil), trypsin 0.25% with ethylenediaminetetraacetic acid (GIBCO^®^, Grand Island, NY, USA), Fetal Bovine Serum (FBS) (GIBCO^®^, Grand Island, NY, USA), BD Phosflow™ Alexa Fluor^®^ 488 mouse anti-AKT (pS473) (BD Biosciences^®^, NJ, EUA), BD Phosflow™ PE mouse anti-NF-κB p65 (pS529) (BD Biosciences^®^, San Jose, CA, USA), BD Phosflow™ PerCP-Cy™5.5 mouse anti-ERK1/2 (pT202/pY204) (BD Biosciences^®^, San Jose, CA, USA), BD Phosflow™ Alexa Fluor^®^ 647 mouse anti-JNK (pT183/pY185) (BD Biosciences^®^, San Jose, CA, USA), BD Phosflow™ PE-Cy™7 mouse anti-p38 MAPK (pT180/pY182) (BD Biosciences^®^, San Jose, CA, USA), U0126 (Sigma-Aldrich^®^; St. Louis, MO, USA), SP600125 (Sigma-Aldrich^®^; St. Louis, MO, USA), and PD 169316 (Sigma-Aldrich^®^; St. Louis, MO, USA).

The drugs and reagent solutions were prepared immediately before use.

### 4.2. Human Tumor Cell Line

The SK-MEL-28 (human melanoma) cell line was obtained from Rio de Janeiro Cell Bank (BCRJ), Brazil, and cultured in Dulbecco’s Modified Eagle’s Medium (DMEM) supplemented with 10% Fetal Bovine Serum and 1% penicillin–streptomycin at 37 °C with 5% CO_2_.

### 4.3. Cell Cycle Analysis

To assess the effect of CBEO on cell cycle progression, SK-MEL-28 cells were stained with PI (propidium iodide) for the DNA content analysis and analyzed by flow cytometry. The fluorescence intensity emitted by PI bound to DNA allows for the quantification of DNA in cells at each phase of the cell cycle [103]. For this purpose, SK-MEL-28 cells were plated (2 × 10^5^ cells/mL) in 24-well plates and incubated with CBEO at concentrations of 20 or 40 µg/mL (corresponding to IC_50_ and double the CI_50_, respectively) or DXR (4 µM) for 48 h. After this period, the cells were collected, centrifuged (500× *g*, 20 °C, 5 min), gently resuspended in PBS, fixed with pre-chilled 70% ethanol while being agitated, and frozen (−20 °C) until the analysis. At the time of the analysis, the cells were recovered by centrifugation (400× *g*, 10 min, 4 °C), washed with PBS, and incubated with RNase (0.1 mg/mL) and propidium iodide (PI; 0.05 mg/mL) in the dark (25 °C, 30 min). Subsequently, the analysis was conducted using a flow cytometer, acquiring 10,000 events/sample. The percentages of cells in different phases of the cell cycle, as well as sub-diploid (sub-G1) cells, were determined. Data were obtained using the DIVA 6.0 software. Three independent experiments were performed in duplicate [104].

### 4.4. Apoptosis Analysis by Flow Cytometry

For the evaluation of the type of cell death induced by CBEO in SK-MEL-28 cells, an Annexin V conjugated with a fluorescein isothiocyanate (FITC) and propidium iodide (PI) staining assay was performed. In this assay, SK-MEL-28 cells were plated (2 × 10^5^ cells/mL) in 24-well plates and incubated with CBEO (20 or 40 µg/mL) or DXR (4 µM) for 48 h. After, the cells were collected and centrifuged (500× *g*, 20 °C, 5 min), they were resuspended in a binding buffer. Subsequently, the cells were labeled with Annexin V–FITC and incubated at room temperature for 10 min in the dark. After this period, the cells were washed with the binding buffer, centrifuged (500× *g*, 20 °C, 5 min), resuspended in the binding buffer, and labeled with PI (20 µg/mL). Then, the analysis was conducted using a flow cytometer, acquiring 10,000 events/sample. Data were obtained using the DIVA 6.0 software. Three independent experiments were performed in duplicate [104].

### 4.5. Apoptosis Analysis by Laser Confocal Microscopy

In this assay, a morphological analysis of SK-MEL-28 cells after CBEO treatment was conducted. The cells were stained with acridine orange (AO) and propidium iodide (PI). SK-MEL-28 cells were plated (2 × 10^5^ cells/mL) in 24-well plates and incubated with CBEO (20 or 40 µg/mL) or DXR (4 µM) for 48 h. Subsequently, the cells were collected and centrifuged (500× *g*, 20 °C, 5 min), resuspended in PBS, and stained with AO (1 mg/mL) and the PI solution (10 μg/mL). The stained cells were observed under a laser scanning confocal microscope. The established criteria were as follows: (a) viable cells exhibited a clear green nucleus with an intact structure; (b) early apoptotic cells displayed a bright green nucleus, indicating chromatin condensation; (c) late apoptotic cells exhibited dense orange areas (stained in both green and red) of chromatin condensation and the formation of apoptotic blebs on the membrane; (d) necrotic or dead cells were stained only in red [105,106,107]. Three independent experiments were performed in duplicate.

### 4.6. Docking Prediction

For the in silico assays, the Molegro Virtual Docker (MVD) v.6.0.1 software was employed (molexus.io/molegro-virtual-docker/). Molecular docking was carried out with enzymes that already had their co-crystallized ligands in their structures: Extracellular Signal-Regulated Kinase 1 (ERK1) (Protein Data Bank (PDB) ID: 5LCJ) and c-Jun N-terminal Kinase 1 (JNK1) (PDB ID: 2G01). Additionally, structures of p38 Mitogen-Activated Protein Kinase α (p38α MAPK) (PDB ID: 1R39), Nuclear Factor *kappa* B (NF-κB; p50/p65) (PDB ID: 1VKX), and Protein Kinase B (PKB/AKT) (PDB ID: 1GZN) were used. In the absence of their ligands in the PDB, catalytic site coordinates were obtained from the literature [73,108,109,110]. The tested molecules were designed using Marvin Sketch v. 19.18 software. Subsequently, the compounds were standardized using ChemAxon’s Standardizer v. 21.2.0 software, which added hydrogen atoms, standardized the aromatic ring, removed salts, converted the structure to 3D, and then subjected the compounds to molecular docking. All water molecules were removed from the crystalline structure. A template was created between the enzyme and the co-crystallized ligand to delineate the active site of the macromolecule. Subsequently, the molecules were added, and the docking prediction was performed. Prior to the docking simulation, redocking was performed, which corresponds to the root mean square deviation (RMSD) calculated from the poses, indicating the degree of confidence in the fit. The RMSD provides a measure of proximity to the experimental structure and is considered successful if the value is less than 2.0 Å [111].

The compounds were imported to analyze the stability of the system through the interactions identified with the active site of the enzyme, taking as a reference the energetic value of the MolDock Score and Rerank Score algorithms [112,113]. The MolDock SE (Simplex Evolution) algorithm was used with the following parameters: a total of 30 runs with a maximum of 3000 interactions using a population of 50 individuals, 2000 minimization steps for each flexible residue, and 2000 minimization steps overall per race. The MolDock Score (GRID) and Rerank Score scoring functions were used to calculate docking energy values. A GRID was set at 0.3 Å and the search sphere was set at a 15 Å radius. To analyze the ligand energy, internal electrostatic interactions, internal hydrogen bonds, and sp2-sp2 torsions were evaluated. To visualize the interactions and obtain the molecular docking figures, the Discovery Studio Visualizer v20.1.0.19295 software was used—BIOVIA (2020) (discover.3ds.com/discovery-studio-visualizer-download) (accessed on 14 August 2023). 

A consensus analysis using two different scoring functions was used to decrease the number of false positives [114]. The results of the affinity of the compounds studied by the MolDock Score and Rerank Score functions were considered in the consensus calculation. Initially, for each of the scoring functions under study, the value of (*p*) was calculated, which is equivalent to dividing the score obtained by each of the compounds by the compound that obtained the lowest energy, according to Equation (1): Prob = (E Lig)/(EMin Lig). Where E Lig is the energy obtained by each ligand in the developed docking simulation, the EMin Lig parameter corresponds to the lowest energy obtained among the ligands under study.

The second consensus analysis refers to the calculation of the general average obtained in all biomarkers under study. In this way, the probability values for each of the compounds under study are calculated as a general average of the probability obtained for each of the biomarkers. After obtaining the probability values obtained for the compounds in each of the scoring functions under study, the total probability (*p*) was calculated, which corresponds to the sum of the probability values obtained for each of the scoring functions. The score under study, divided by the total number of observations, according to Equation (2), is: p Enzyme = (p MolDock Score + p Rerank Score)/n.

### 4.7. MAPK Signaling Analysis by Flow Cytometry

To investigate the effect of CBEO on the modulation of these proteins in SK-MEL-28 cells, the cells were plated (2 × 10^5^ cells/mL) in 24-well plates. After 24 h, the cells were incubated with CBEO (20 or 40 µg/mL) or DXR (4 µM) for 48 h. After this time, the cells were collected, centrifuged (500× *g*, 20 °C, 5 min), and fixed for 10 min at 37 °C. Subsequently, the cells were washed and permeabilized. After this procedure, the cells were resuspended and labeled with anti-ERK1/2, anti-JNK, or anti-p38 MAPK antibodies, following the manufacturer’s recommendations. After 30 min of incubation with the antibodies (25 °C, in the dark), the cells were washed and centrifuged (300× *g*, 4 °C, 5 min), and the supernatant was removed. Then, the cells were resuspended once more, and the analysis was performed using a flow cytometer, acquiring 10,000 events/sample. Data were obtained using the DIVA 6.0 software. Three independent experiments were conducted in duplicate.

### 4.8. Evaluation of CBEO Cytotoxicity in the Presence or Absence of MAPKs Inhibitors

To assess the involvement of MAPKs in CBEO cytotoxicity, the MTT assay was conducted in the presence or absence of MAPK inhibitors. SK-MEL-28 cells were plated (96-well plates; 3 × 10^5^ cells/mL) and incubated for 24 h at 37 °C, CO_2_ 5%. Subsequently, the cells were incubated in the presence or absence of the 40 μM ERK inhibitor (U0126), 20 μM JNK inhibitor (SP600125), or 20 μM p38 MAPK inhibitor (PD 169316) for 3 h. Subsequently, the cells were treated with CBEO (20 µg/mL) or DXR (4 µM) and incubated for 72 h (CO_2_ 5%; 37 °C). The plates were centrifuged, and the supernatant was removed. Then, 10 µL of the MTT solution (5 mg/mL) was added, and the plate was further incubated for 4 h in an atmosphere of 5% CO2 at 37 °C. After this incubation period, 100 μL of SDS [115] was added to solubilize the formazan crystals. Optical density was determined using a microplate reader (Synergy HT, BioTek^®^, Winooski, VT, EUA) at λ = 570 nm. Three independent experiments were conducted in triplicate.

### 4.9. NF-κB and PKB/AKT Signaling Analysis by Flow Cytometry

To investigate the effect of CBEO on the modulation of these signaling pathways in SK-MEL-28 cells, the cells were plated (2 × 10^5^ cells/mL) in 24-well plates. After 24 h, the cells were incubated with CBEO (20 or 40 µg/mL) or DXR (4 µM) for 48 h. After this time, the cells were collected, centrifuged (500× *g*, 20 °C, 5 min), and fixed for 30 min at 4 °C. Subsequently, the cells were permeabilized (30 min on ice). After, the cells were washed, resuspended, and labeled with anti-NF-κB/p65 or anti-PKB/AKT antibodies, following the manufacturer’s recommendations. After 30 min of incubation (25 °C, in the dark), the cells were washed, centrifuged (300× *g*, 4 °C, 5 min), and the supernatant was removed. Then, the cells were resuspended, and the analysis was performed using a flow cytometer, acquiring 10,000 events/sample. Data were obtained using the DIVA 6.0 software. Three independent experiments were performed in duplicate.

### 4.10. Statistical Analysis

The statistical analysis was performed using GraphPad Prism 8.0.2 (Graphpad Software Inc., San Diego, CA, USA). Results are expressed as the mean ± standard error of the mean (SEM). The data statistical analysis was performed using Analysis of Variance (ANOVA) one-way, followed by Tukey’s test (*p* < 0.05).

## 5. Conclusions

Preliminary data obtained by Ferreira et al. (2023) [19] have demonstrated that the in vitro antimelanoma effect of CBEO is Reactive Oxygen Species-dependent. Oxidative stress is implicated in the modulation of various intracellular signaling pathways, including MAPKs, NF-κB, and PKB/AKT. Herein, our results provide evidence of the involvement of these signaling pathways in the apoptosis induced by CBEO on SK-MEL-28 cells. Thus, CBEO represents a promising therapeutic alternative for melanoma treatment. Hence, additional research is required to enhance our comprehension of the mode of action of this EO.

## Figures and Tables

**Table 1:** Binding-energy (kJ/mol) values of (Z)-2-lachnophyllum ester (EZ), doxorubicin (DXR), or other ligands with Mitogen-Activated Protein Kinases (MAPKs), Nuclear Factor *kappa* B (NF-κB), and Protein Kinase B (PKB/AKT).

Protein	Ligand	MolDock Score *	(p) ^†^ MolDock Score	Rerank Score *	(p) ^†^ Rerank Score	(p) ^†^ Total
ERK1 ^a^	EZ	−63.2634	0.5106	−52.153	0.7704558	0.6405279
	DXR	−110.085	0.8886	−67.6911	1	0.9443
	PDBL ^f^	−123.881	1	−66.5335	0.9828988	0.9914494
JNK1 ^b^	EZ	−70.0612	0.5941	−61.538	0.7443	0.6692
	DXR	−117.919	1	−82.6781	1	1
	PDBL ^g^	−67.8571	0.5754	−57.9951	0.7014	0.6384
p38α MAPK ^c^	EZ	−76.5248	0.739384	−62.4631	0.987851	0.863618
	DXR	−100.213	0.96826	−63.2313	1	0.98413
	CTL ^h^	−103.498	1	−59.1131	0.934871	0.967435
NF-κB (p50/p65) ^d^	EZ	−83.0295	0.720536	−69.7697	0.844223	0.782379
	DXR	−115.233	1	−82.6437	1	1
	CTL ^i^	−100.634	0.873309	15.8197	0	0.436654
PKB/AKT ^e^	EZ	−107.102	0.727012	−90.3473	0.806392	0.766702
	DXR	−142.547	0.967614	−112.039	1	0.983807
	CTL ^j^	−147.318	1	−83.2668	0.743195	0.871597

^a^ ERK1: Extracellular Signal-Regulated Kinase 1; ^b^ JNK1: c-Jun N-terminal Kinase 1; ^c^ p38α MAPK: p38 Mitogen-Activated Protein Kinase α (p38α MAPK); ^d^ NF-κB; p50/p65: Nuclear Factor *kappa* B; ^e^ PKB/AKT: Protein Kinase B; ^f^ PDBL (Protein Data Bank ligand): [(1R,4Z)-cyclooct-4-en-1-yl]-N-[4-[4-[[5-chloro-4-[[2-(propanoylamino)phenyl]amino]pyrimidin-2-yl]amino]pyridin-2-yl]but-3-inyl]carbamate; ^g^ PDBL (Protein Data Bank ligand): 6-chloro-9-hydroxy-1,3-dimethyl-1,9-dihydro-4H-pyrazolo(3,4-b)quinolin-4-one; ^h^ CTL (Control ligand of p38α MAPK): (4-(4-fluorophenyl)-2-(4-methylsulfinylphenyl)-5-(4-pyridyl)-imidazole); ^i^ CTL (Control ligand of NF-κB (p50/p65)): 4-methyl-N1-(3-phenylpropyl)benzene-1,2-diamine; ^j^ CTL (Control ligand of PKB/AKT): 8-[4-(1-aminocyclobutyl)phenyl]-9-phenyl[1,2,4]triazolo[3,4-f][1,6]naphthyridin-3(2H)-one; * Energy values expressed in kJ/mol; ^†^ Affinity probability value.

## Data Availability

Data is contained within the article and supplementary material.

## Supplementary Materials

The following supporting information can be downloaded at: https://www.mdpi.com/article/10.3390/ph16111553/s1, Figure S1: Chromatogram of the essential oil from *Conyza bonariensis* (L.) aerial parts (CBEO); and Figure S2: ^1^H NMR spectrum of the essential oil from *Conyza bonariensis* (L.) aerial parts (CBEO).
