# Supplementary material for: The Essential Oil from Conyza bonariensis (L.) Cronquist (Asteraceae) Exerts an In Vitro Antimelanoma Effect by Inducing Apoptosis and Modulating the MAPKs, NF-κB, and PKB/AKT Signaling Pathways
Source: Pharmaceuticals (Basel). 2023 Nov 2;16(11):1553. doi: 10.3390/ph16111553 (PMC10674350; doi:10.3390/ph16111553)
Supplement: Supplementary file 1 [file pharmaceuticals-16-01553-s001.zip › pharmaceuticals-2674692-supplementary.pdf]

SUPPORTING INFORMATION

The Essential Oil from *Conyza bonariensis* (L.) Cronquist (Asteraceae) Exerts In Vitro Antimelanoma Effect by Inducing Apoptosis and Modulating the MAPKs, NF-κB, and PKB/AKT Signaling Pathways

Rafael Carlos Ferreira [1], Sâmia Sousa Duarte [1], Valgrícia Matias de Sousa [1], Ramon Ramos Marques de Souza [1], Karinne Kelly Gadelha Marques [1], Renata Albuquerque de Abrantes [1], Yuri Mangueira do Nascimento [1], Natália Ferreira de Sousa [1], Marcus Tullius Scotti [1], Luciana Scotti [1], Josean Fechine Tavares [1], Juan Carlos Ramos Gonçalves [1], Marcelo Sobral da Silva [1] and Marianna Vieira Sobral [1,*]

[1] Postgraduate Program in Natural Products and Bioactive Synthetics, Federal University of Paraíba, João Pessoa 58051-970, PB, Brazil
* Correspondence: mariannavbs@gmail.com

CONTENT

Figure S1. Chromatogram of the essential oil from *Conyza bonariensis* (L.) aerial parts (CBEO)....3

Figure S2. ^{1}H NMR spectrum of the essential oil from *Conyza bonariensis* (L.) aerial parts (CBEO) 3

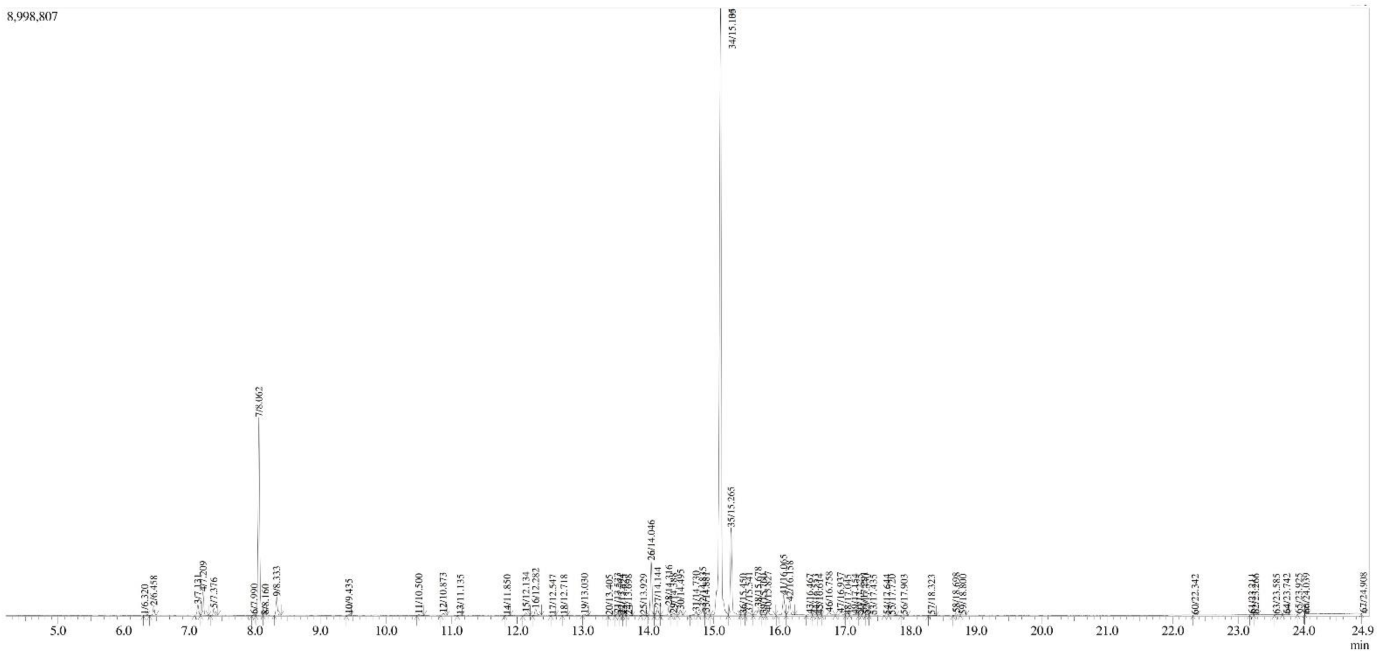

Figure S1. Chromatogram of the essential oil from *Conyza bonariensis* (L.) aerial parts (CBEO)

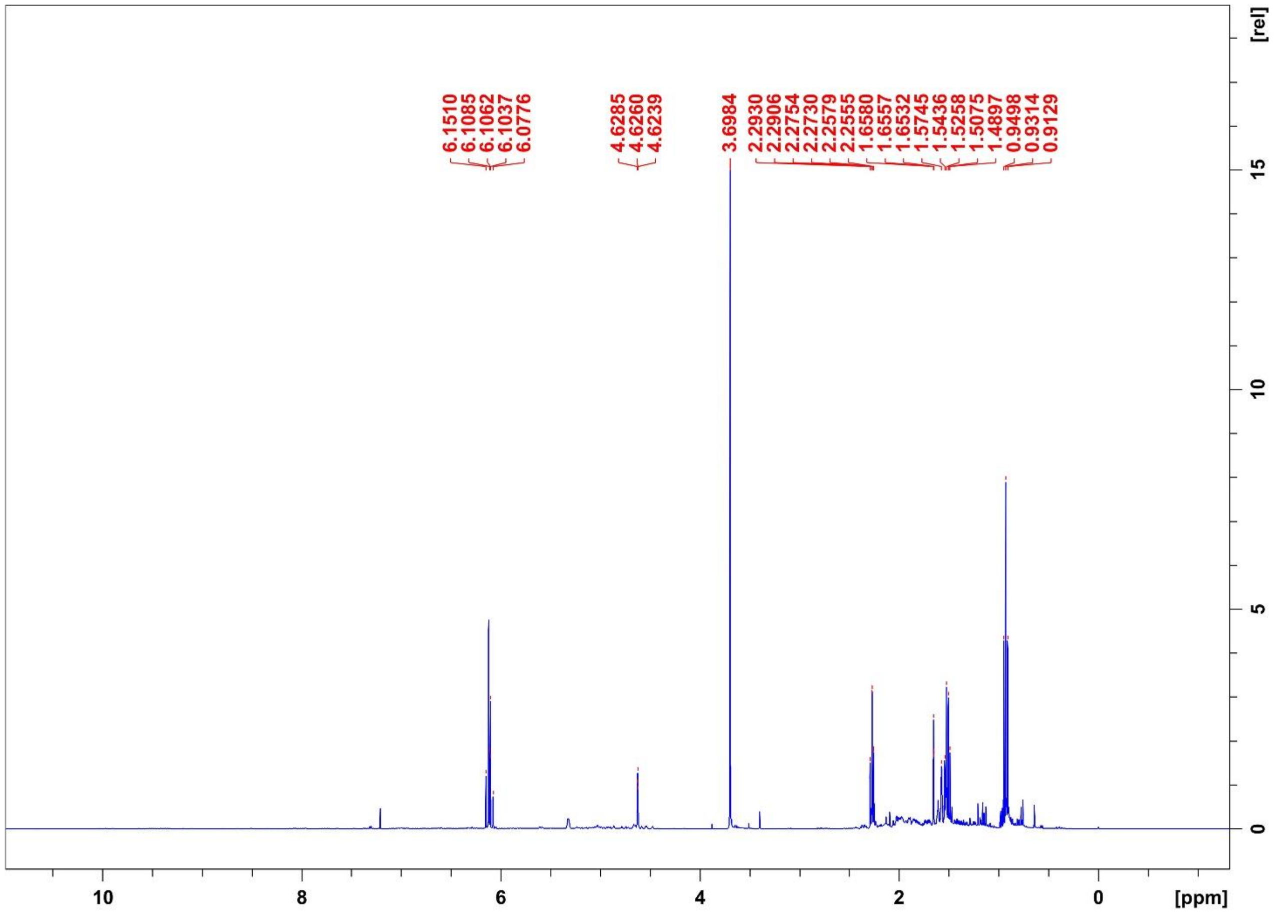

Figure S2. ¹H NMR spectrum of the essential oil from *Conyza bonariensis* (L.) aerial parts (CBEO)
